# Complete Genome Sequence of *Pseudomonas denitrificans* ATCC 13867

**DOI:** 10.1128/genomeA.00257-13

**Published:** 2013-05-30

**Authors:** Satish Kumar Ainala, Ashok Somasundar, Sunghoon Park

**Affiliations:** Department of Chemical and Biomolecular Engineering, Pusan National University, Geumjeong-gu, Busan, South Korea

## Abstract

*Pseudomonas denitrificans* ATCC 13867, a Gram-negative facultative anaerobic bacterium, is known to produce vitamin B_12_ under aerobic conditions. This paper reports the annotated whole-genome sequence of the circular chromosome of this organism.

## GENOME ANNOUNCEMENT

Vitamin B_12_ is one of the most complex nonpolymeric biological molecules (1). It is essential for the proper function of the animal nervous system (2) and is used as a coenzyme for many microbial enzymes, including ethanolamine ammonia lyase, methionine synthase, diol dehydratase, and glycerol dehydratase (3–5). However, only a few microorganisms, such as *Pseudomonas denitrificans, Klebsiella pneumoniae, Salmonella enterica* subsp. *enterica* serovar Typhimurium, *Propionibacterium freudenreichii*, and *Bacillus megaterium*, can produce this important vitamin (6–8). Most microorganisms produce vitamin B_12_ under anaerobic conditions, while *P. denitrificans* produces B_12_ under aerobic conditions. *P. denitrificans* has been exclusively used for the industrial production of this vitamin (9). This paper reports the whole-genome sequence of *P. denitrificans* ATCC 13867, with an emphasis on its synthesis of vitamin B_12_.

The complete genome of *P. denitrificans* ATCC 13867 was sequenced using the 454 GS FLX Titanium system (Roche Diagnostics, Switzerland), with a combination of the 8-kb mate-pair library (623,018 reads; length, 231,129,016 bp) and the shotgun single-read library (653,245 reads; length, 262,508,692 bp) (10). The sequences obtained from these combined approaches were assembled using a Newbler assembler (Roche Diagnostics, Switzerland), which showed 45.78-fold coverage. The genome size of *P. denitrificans* ATCC 13867 is 5,696,307 bp with 65.2% G+C content. Gene prediction and annotations were carried out using the Basic Local Alignment Search Tool and an automated annotation system, Ensoltek (11). The *P. denitrificans* ATCC 13867 genome has 2,567 operons and 5,059 protein-encoding genes. It has genes for the 62 transfer RNAs (tRNAs), which represent all 20 amino acids. The ribosome-binding sites (RBS) and transcriptional terminators were predicted using the RBS finder and TransTerm tools, respectively (12). The prediction suggested 1,279 RBS and 816 transcription terminators. The protein-coding genes were subjected to the PSORTb protein tool to identify the subcellular localization of the proteins (13). Among the 5,059 proteins, 3,013 (59.56%) proteins were expected to be cytoplasmic and 982 proteins (19.41%) were noncytoplasmic. The locations of the remaining 1,045 (21.02%) proteins could not be identified.

The essential amino acid l-methionine is synthesized by two different pathways, coenzyme B_12_-independent (*metE*, cobalamin-independent homocysteine transmethylase) and coenzyme B_12_-dependent (*metH*, methionine synthase) (14). The *P. denitrificans* genome had only *metH*, indicating that coenzyme B_12_ is essential for the synthesis of l-methionine (15). The genome of *P. denitrificans* contains all the genes (encoding 26 enzymes) necessary for oxygen-dependent coenzyme B_12_ synthesis. The genes are distributed in two different clusters on the chromosome (clusters I and II). Cluster I consists of 5 operons that encode nine enzymes required for the formation of hydrogenobyrinic acid, an important intermediate of coenzyme B_12_, from uroporphyrinogen III. Operon 1 of this cluster contains *cobGHIJ*, operon 2 contains *cobLFK*, and operon 3 contains *chlLD* and *xre/pbsX*. On the other hand, operon 4 contains *cobWN*, and operon 5 contains *cbtBA* and *cobEM*. The genes *cobOBRDCQPUV*, *bpgM* (encoding bisphosphoglycerate mutase), and *tonB*, which are involved in the conversion of hydrogenobyrinic acid to adenosylcobalamine (16), were located separately as a single operon on cluster II.

### Nucleotide sequence accession number.

The *P. denitrificans* ATCC 13867 whole-genome sequence assembly and its annotation were deposited in GenBank under the project accession no. CP004143.
